# Primary hyperparathyroidism

**DOI:** 10.1002/ccr3.2035

**Published:** 2019-03-05

**Authors:** Ao Wang, Lei Yuan

**Affiliations:** ^1^ Department of Endocrinology, the Second Affiliated Hospital Chongqing Medical University Chongqing China

**Keywords:** emission computed tomography, hypercalcemia, hyperparathyroidism, parathyroid adenoma

## Abstract

We report a case of hyperparathyroidism with hypercalcemia caused by a giant parathyroid adenoma. We use a technetium sestamibi scan to locate parathyroid mass. Bone emission computed tomography revealed the classic signs of metabolic osteopathy. She was recovered after parathyroidectomy.

A 54‐year‐old woman presented to Endocrine department with polydipsia, polyuria, nausea, and anorexia over 2 months. She had a 10‐year history of renal calculus. Laboratory evaluation revealed a hypercalcemia at 3.47 mmol/L (reference range, 2.12‐2.92), hypophosphatemia at 0.65 mmol/L (reference range, 0.85‐1.51). The plasma parathyroid hormone (PTH) level was 592.66 pg/L (reference range, 6.50‐36.80). Notably, the plasma alkaline phosphatase level was 389 U/L (reference range, 50‐135). Neck ultrasonography showed a mixed echo mass measuring 5.0 by 3.2 cm in the right thyroid area. Parathyroid emission computed tomography (ECT) demonstrated a large of nuclide intake under the right sublobe of the thyroid gland (Figure 1A). Whole‐body bone ECT showed a metabolic bone "super scan" (Figure 1B). A super scan is defined as intense symmetric activity in the bones and diminished renal parenchymal activity. The patient was diagnosed with primary hyperparathyroidism. About 80% of primary hyperparathyroidism is due to a single benign tumor known as a parathyroid adenoma. The diagnosis was confirmed by parathyroidectomy and histopathology. After operation, the patient recovered with normalization of serum calcium and PTH.

**Figure 1:**
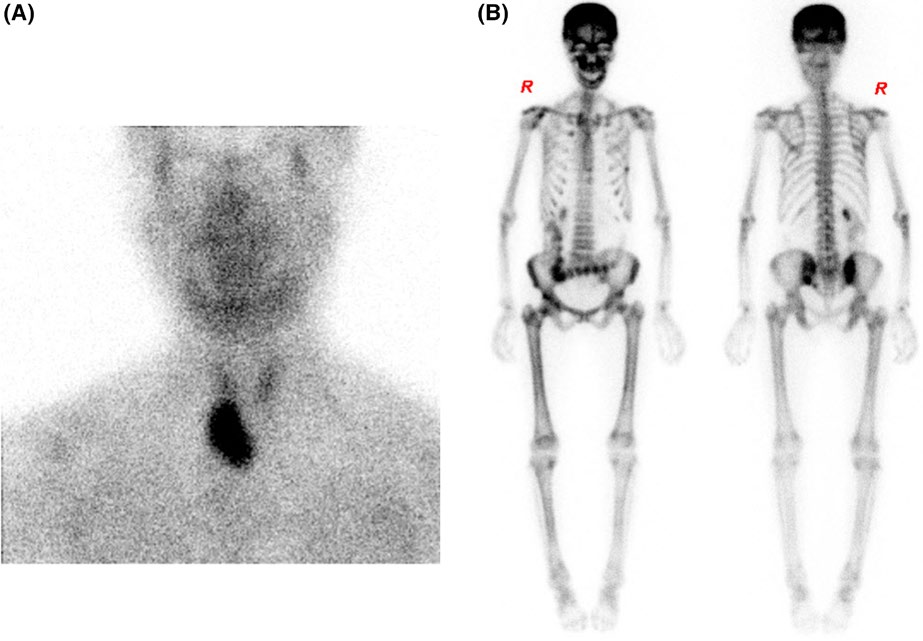
Parathyroid emission computed tomography (A) and whole‐body bone imaging emission computed tomography (B)

## CONFLICT OF INTEREST

Ao Wang and Lei Yuan declare that they have no conflict of interest.

## AUTHOR CONTRIBUTION

AW: mainly contributed to the data collection. LY: drafted the manuscript and taken responsibility for this work.

